# Association of TLR3 (rs3775291) and IL-10 (rs1800871) gene polymorphisms with susceptibility to Hepatitis B infection: A meta-analysis

**DOI:** 10.1017/S0950268820002101

**Published:** 2020-09-11

**Authors:** Susu Ye, Xinlei Zhang, Yu bao Zhang, Xintao Tian, Ailing Liu, Changxing Cui, Lei Shi, Di Xia

**Affiliations:** 1 Liver Disease Center, The Affiliated Hospital of Qingdao University, Qingdao, Shandong, China; 2 Department of Medical Engineering, The 971st Hospital of the PLA, Qingdao, Shandong, China; 3 Department of Emergency, The Affiliated Hospital of Qingdao University, Qingdao, Shandong, China; 4 Department of Gastroenterology, The Affiliated Hospital of Qingdao University, Qingdao, Shandong, China

**Keywords:** Hepatitis B virus, Interleukin 10, meta-analysis, polymorphism, Toll-like receptor 3

## Abstract

TLR3 and IL-10 play a crucial role in antiviral defence. However, there is a controversy between TLR3 rs3775291 and IL-10 rs1800871 polymorphisms and the risk of hepatitis B virus (HBV) infection. The purpose of this study is to explore the relationship between the two single nucleotide mutations and the risk of HBV infection by meta-analysis. Medline, EMBASE, Web of Science, CNKI, China Wanfang database were searched for the case-control studies on the relationship between TLR3 rs3775291 and IL-10 rs1800871 polymorphism and susceptibility to HBV, updated to June 2020. The data were analysed by Stata 15.0 software. A total of 22 articles were included. The results showed that in the analysis of IL10 rs1800871 polymorphism and the risk of HBV infection, the pooled OR was 1.21 (95% CI 1.06–1.37), 1.28 (95% CI 1.04–1.56) and 1.20 (95% CI 1.06–1.37) and 1.40 (95% CI 1.07–1.83) in the allele model (C *vs.* T), dominant model (CC+CT *vs.* TT), recessive model (CC *vs.* CT+TT) and homozygous model (CC *vs.* TT), respectively. There was no statistical significance in the heterozygote model. A subgroup analysis of the Asian population showed similar results. The analysis of TLR3 rs3775291 polymorphism and the risk of HBV showed that in the allele model (T *vs.* C), the pooled OR was 1.30 (95% CI 1.05–1.61). Except for the recessive model, no significances were found in other genetic models. In conclusion, TLR3 rs3775291 and IL-10 rs1800871 polymorphisms are associated with the risk of HBV. Allele C and genotype CC at IL10 rs1800871 loci, as well as allele T and genotype TT at TLR rs3775291 loci, may increase susceptibility to Hepatitis B infection.

## Introduction

Hepatitis B is a kind of infectious disease caused by the hepatitis B virus (HBV), mainly caused by liver inflammation and can cause pathological damage such as liver cirrhosis and liver cancer. It is widely prevalent worldwide, especially in developing countries and brings great harm to human health [1, 2]. HBV infection can lead to self-limited, acute, chronic viral hepatitis, liver cirrhosis, liver cancer and other serious liver diseases. Most of the infected people can remove the virus self-limitedly and if the infection persists, it will develop into chronic hepatitis B [3]. The host immune system, including innate immune response and adaptive immune response, plays a crucial role in the process of HBV infection. Besides, viral factors, host factors and environmental factors can affect the outcome of HBV infection [4–6]. In recent years, the study of host genetic susceptibility has attracted wide attention.

Toll-like receptor (TLR), one of the pathogen pattern recognition receptors, is a transmembrane protein receptor on the surface of mammalian cells. TLR3 mainly recognises double-stranded RNA related to virus infection and virus replication and induces apoptosis in infected cells [7]. TLR3 is located on the 4q35 chromosome and encodes a total of 904 amino acids. The primary gene polymorphism site rs3775291 (C1234T): T mutated from C on exon 4, which causes the 421 leucine to be replaced by phenylalanine, which is associated with encephalitis, HIV, HCV and other diseases [8, 9]. This single nucleotide polymorphism (SNP) is thought to play a vital role in the pathological process of HBV, which may be associated with the risk of HBV infection [10]. Moreover, the level of TLR3 in patients with active chronic hepatitis B was significantly higher than that in healthy controls. At present, the relationship between TLR3 rs3775291 polymorphism and HBV is still controversial. Rong *et al*. [11] and Fischer *et al*. [12] reported that allele T at the TLR3 rs3775291 loci increased the risk of HBV infection. However, other studies [13, 14] suggested that its allele T was not related to the risk of HBV infection.

IL-10, an important Th2 cytokine, plays an essential regulatory role in the immune process of HBV infection. The increase of serum IL-10 levels after HBV infection limits the inflammatory response that causes tissue injury and becomes a key regulator of the immune system [15]. IL-10 can inhibit the secretion of cytokines TNF- *α* and IFN- *γ* secreted by Th1 and has a crucial antiviral function. The secretion level of IL-10 depends to a large extent on genetic factors. The risk of HBV infection is closely related to IL-10-819C/T (rs1800871) polymorphism, but there are vast differences in different studies. Some studies [16, 17] suggested that allele C at the IL-10 rs1800871 loci increased the risk of HBV infection. However, some studies [18, 19] suggested that allele C at the IL-10 rs1800871 site was not associated with the risk of HBV infection.

It can be seen that the relationship between TLR3 rs3775291 and IL-10 rs1800871 polymorphisms and HBV is controversial. Therefore, this study uses meta-analysis to explore the association between the two single nucleotide polymorphisms and susceptibility to HBV infection, exploring the pathogenicity of HBV at Gene level, to provide new ideas for finding gene therapy targets.

## Methods

### Retrieval strategy

Case-control studies on the relationship between TLR3 and IL-10 gene polymorphisms and HBV infection were searched in Medline (via PubMed) (January 1950 to June 2020), Excerpta Medica Database (EMBASE) (January 1974 to June 2020), Web of Science (January 1945 to June 2020), China national knowledge infrastructure (CNKI) (June 1999 to June 2020) and China Wanfang database (January 1998 to June 2020). The search strategy is as follows: (‘Hepatitis B’ OR ‘chronic hepatitis B’ OR ‘hepatitis B virus’ OR ‘HBV’) AND (‘TLR3’ OR ‘Toll-like receptor 3’ OR ‘IL-10’ OR ‘interleukin 10’) AND (‘polymorphism’ OR ‘single nucleotide polymorphism’ OR ‘SNP’). The languages included in the study are limited to Chinese and English. If there were multiple publications with the same theme, we used the most complete and up-to-date results. A reference list of selected articles (including comments) was also reviewed to identify other relevant publications.

### Inclusion and exclusion criteria

#### Inclusion criteria

(1) Case-control studies on the relationship between TLR3 rs3775291 and IL-10 rs1800871 polymorphisms and the risk of hepatitis B infection; (2) human beings as the object of study; (3) the original data provided in the articles can be extracted or used to calculate the genotype frequencies of TLR3 rs3775291 and IL-10 rs1800871 gene polymorphisms; (4) only published full-text studies were included.

#### Exclusion criteria

(1) Letters, abstracts, and minutes of scientific meetings addressed to editors; (2) studies that do not meet the current inclusion criteria; (3) unpublished data sources were not included in this study; (4) Newcastle-Ottawa Scale (NOS) score was less than 6; (5) the frequency of genotypes in the control group did not meet Hardy-Weinberg equilibrium (HWE).

### Data extraction

Two researchers (CCX and SL) evaluated the studies to determine whether they needed to be included and separately evaluated the methodological portion of each study based on inclusion and exclusion criteria. They independently extracted and cross-checked the data and when they encountered differences, turned to the third researcher (YSS) for help. The following information was derived from each study that included: first author, year of publication, country, the ethnic identity of the patient group, number of cases and controls, control sources and genotype frequency.

### Document quality evaluation

According to NOS [20], full texts were carefully read and evaluated. Low-quality literature was those fewer than six stars, while high-quality literature were those with six or more stars. Only those with six or more than six stars were selected. According to the uniform quality standard, the evaluation was conducted independently by two evaluators and then cross-checked. Encountering differences, they resolved them through discussion or turned to the third author for decision.

### Statistical analysis

The fixed-effects model (FEM) or random-effects model (REM) was used to calculate the pooled odds ratio (OR) and its 95% CI to evaluate the correlation between TLR3 rs3775291 and IL-10 rs1800871 gene polymorphisms and the risk of hepatitis B infection. The data were analysed by Stata 15.0 statistical software. Q-test was used to verify the heterogeneity among the studies included [21]. If *I*^2^ ⩾ 50% or *P* ⩽ 0.05, it was considered that there was heterogeneity among studies and the DerSimonia-Laird method was adopted to calculate random effect summary OR [22]. If *I*^2^ < 50% and *P* > 0.05, heterogeneity among studies was considered to be low and the Mantel-Haenszel method was used to calculate fixed effect summary OR [23]. The publication bias was access by the funnel plot and Egger's Test [24]. If the funnel plot was asymmetric, or *P* value of Egger's Test was lower than 0.05, there might be publication bias. The genotype frequency of the control group was tested for HWE by the chi-square test. As there were few studies on the relationship between TLR3 rs3775291 polymorphism and HBV risk, no funnel plot was conducted and only Egger's Test was used to determine the publication bias. The relationship between IL-10 rs1800871 polymorphism and HBV risk was studied and the source of the control group and Ethnicity were analysed by subgroup analysis. Finally, the results of the sensitivity were analysed to explore the robustness of the conclusions. *P* < 0.05 was considered to be statistically significant.

## Results

### Results of literature retrieval

**Fig. 1.**
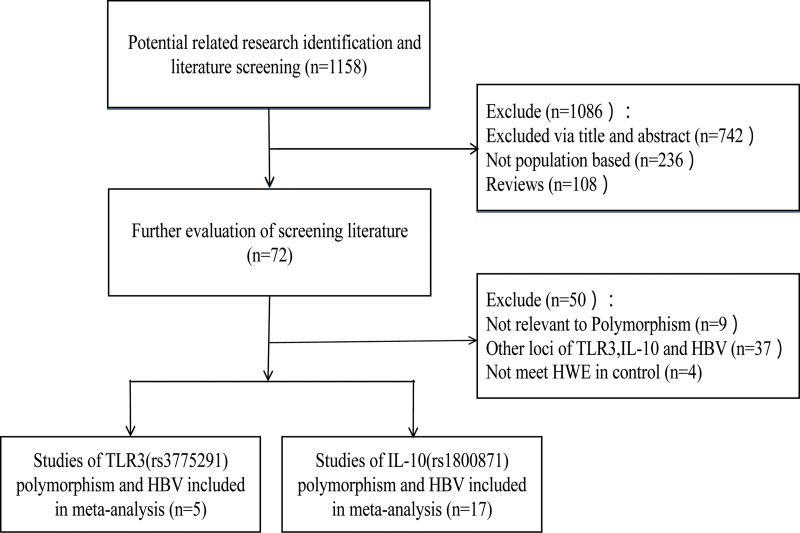
Screening flow diagram for inclusion in the study.

According to the inclusion and exclusion criteria, 22 articles were included in this study, including five articles on TLR3 rs3775291 polymorphism and the risk of HBV [10–14] and 17 articles on IL-10 rs1800871 and susceptibility to HBV [16–19, 25–37]. The detailed literature screening process was shown in Figure 1. The basic characteristics and the NOS scores of the studies included the relationship between TLR3 rs3775291 polymorphism and the risk of HBV were found in Table 1. The basic characteristics of IL-10 rs1800871 polymorphism and hepatitis B included in the study and the results of the literature quality score can be found in Table 2. The basic characteristics and the NOS scores of the studies included on the relationship between IL-10 rs1800871 Single nucleotide polymorphism and the susceptibility to HBV were exhibited in Table 2.

**Table 1.** Basic characteristics of the studies on the correlation between TLR3 rs3775291 polymorphism and HBV

First author	Year	Country	Control source	HBV			Control			*P* for HWE (control)	NOS score
				TT	CT	CC	TT	CT	CC		
Li GG	2013	China	H	41	174	133	11	48	59	0.785	8
Rong YH	2013	China	H	42	212	198	23	189	250	0.091	8
Sá	2015	Brasil	H	3	18	26	33	132	134	0.954	8
Chen DN	2017	China	S	45	150	97	92	323	271	0.784	8
Fischer	2018	German	S	137	236	248	23	92	124	0.332	8

HBV, Hepatitis b virus; H, healthy controls; S, self-limiting infection controls; HWE, Hardy-Weinberg equilibrium; NOS, Newcastle-Ottawa Scale.

**Table 2.** Basic characteristics of the studies on the correlation between IL10 rs1800871 polymorphism and HBV

First author	Year	Country	Control source	HBV			Control			*P* for HWE (control)	NOS score
				CC	CT	TT	CC	CT	TT		
Miyazoe	2002	Japan	H	27	91	95	6	20	26	0.483	8
Li C	2006	China	H	23	81	78	8	21	34	0.119	8
Zhang PA	2006	China	H	25	103	103	12	67	56	0.199	7
Xie HY	2008	China	H	15	93	78	10	68	73	0.266	8
Truelove a	2008	African-Americans	S	18	23	4	24	37	15	0.914	8
Truelove b	2008	European-Americans	S	109	60	10	182	112	19	0.750	8
Yan Z	2009	China	H	120	428	531	33	150	231	0.219	8
Liu JJ	2010	China	H	36	200	183	20	92	75	0.292	8
Qiu XQ	2011	China	H	538	406	117	181	143	35	0.389	8
Wang S	2011	China	H	4	12	36	4	12	33	0.085	8
Wang CJ	2011	China	H	33	168	134	18	71	76	0.817	7
Sodsai	2012	Thailand	H	10	74	47	16	59	67	0.584	8
Wang C	2012	China	H	12	34	16	69	251	205	0.567	8
Sofian	2013	Iran	H	45	43	12	16	11	4	0.358	8
Srivastava	2014	India	H	68	111	53	9	29	38	0.352	8
Talaat	2014	Egypt	H	69	40	6	62	52	5	0.143	8
Chen W	2018	China	H	39	72	104	11	63	46	0.106	7
Rybicka	2020	France	S	182	106	32	6	10	8	0.431	8

HBV, Hepatitis b virus; H, healthy controls; S, self-limiting infection controls; HWE, Hardy-Weinberg equilibrium; NOS, Newcastle-Ottawa Scale.

### Results of meta-analysis of TLR3 rs3775291 polymorphism and risk of HBV infection

**Fig. 2.**
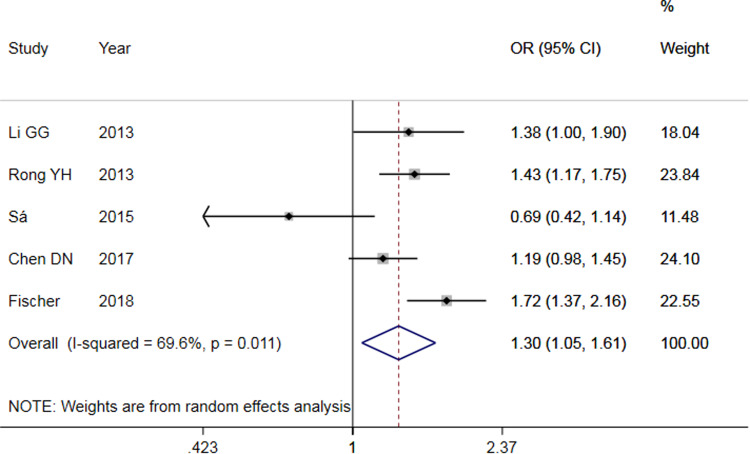
Forest plot of allele model of TLR3 (rs3775291) loci and susceptibility to HBV.

The results of meta-analysis of TLR3 rs3775291 polymorphism and the risk of HBV infection (Table 3) showed that in the allele genetic model (T *vs.* C), dominant genetic model (TT + TC *vs.* CC), recessive genetic model (TT *vs.* TC + CC), homozygous genetic model (TT *vs.* CC), heterozygous genetic model (TC *vs.* CC), the pooled OR was 1.30 (95% CI 1.05–1.61, *P* < 0.05), 1.42 (95% CI 1.23–1.65, *P* < 0.01), 1.53 (95% CI 1.00–2.34, *P* > 0.05), 1.74 (95% CI 1.11–2.73, *P* < 0.05), 1.32 (95% CI 1.13–1.54, *P* < 0.01), respectively. It can be seen that except for the recessive gene genetic model, in which no statistical significance existed, there was all statistical significance in other genetic models. The forest plot of the allele model was shown in Figure 2. Among them, the heterogeneity of the dominant genetic model and the heterozygous genetic model was low. Accordingly, the FEM was used to combine OR. There was great heterogeneity in other genetic models and thus REM was chosen to analyse the data. Due to the small number of studies included, funnel plot detection was not carried out. The results of Egger's Test showed that in each genetic model, the *P-*value was greater than 0.05, indicating that the publication bias was well controlled. This suggested a correlation between TLR3 rs3775291 single nucleotide variation and the risk of HBV infection.

**Table 3.** Main results of meta-Analysis of HBV susceptibility to TLR3 rs3775291 Polymorphisms

Genetic model	*n*	OR	95% CI	*p*	I2	*P* for Heterogeneity	Statistical models	*P* for Publication bias (Egger)
Allelic model	5	1.3	1.05–1.61	0.014	69.6	0.011	REM	0.457
Dominant model	5	1.42	1.23–~1.65	0.000	47.5	0.107	FEM	0.280
Recessive model	5	1.53	1.00–2.34	0.051	62.8	0.029	REM	0.727
Homozygous model	5	1.74	1.11–2.73	0.016	63.3	0.028	REM	0.612
Heterozygous model	5	1.32	1.13–1.54	0.000	15.1	0.318	FEM	0.378

OR, odds ratio; CI, confidence interval; REM, random-effects model; FEM, fixed-effect model.

### The results of meta-analysis of IL-10 rs1800871 polymorphism and the risk of HBV infection

#### Comparison of allelic model

**Fig. 3.**
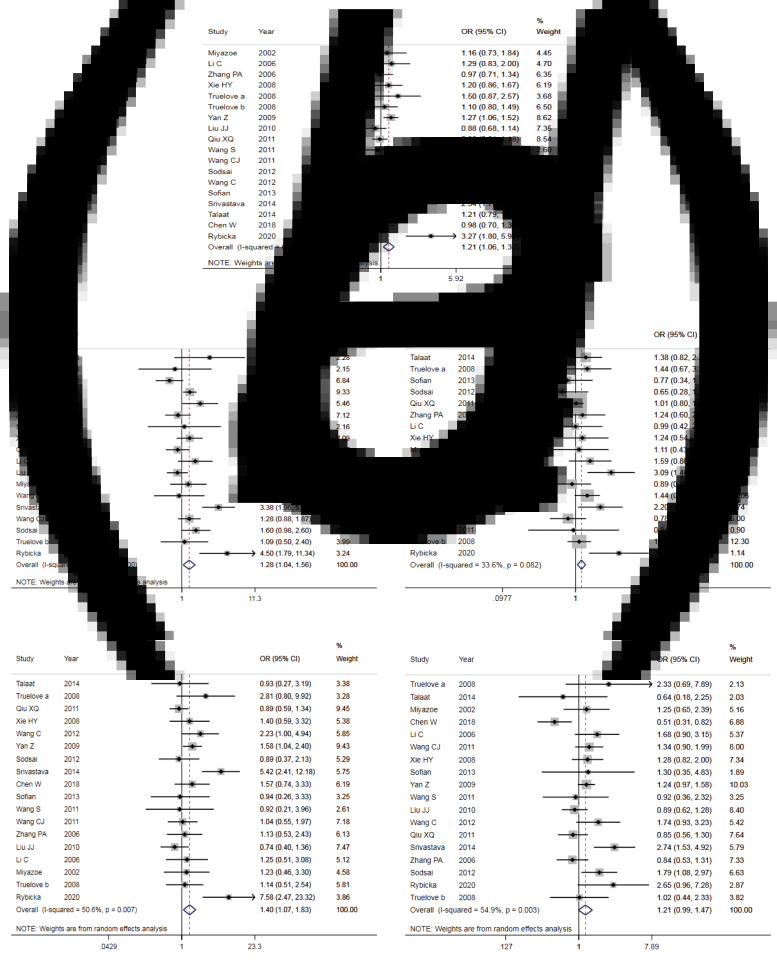
Forest plot of IL-10 (rs1800871) polymorphism and susceptibility to HBV (a: allelic genetic model; b: dominant genetic model; c: recessive genetic model; d: homozygous genetic model; e: heterozygous genetic model).

**Fig. 4.**
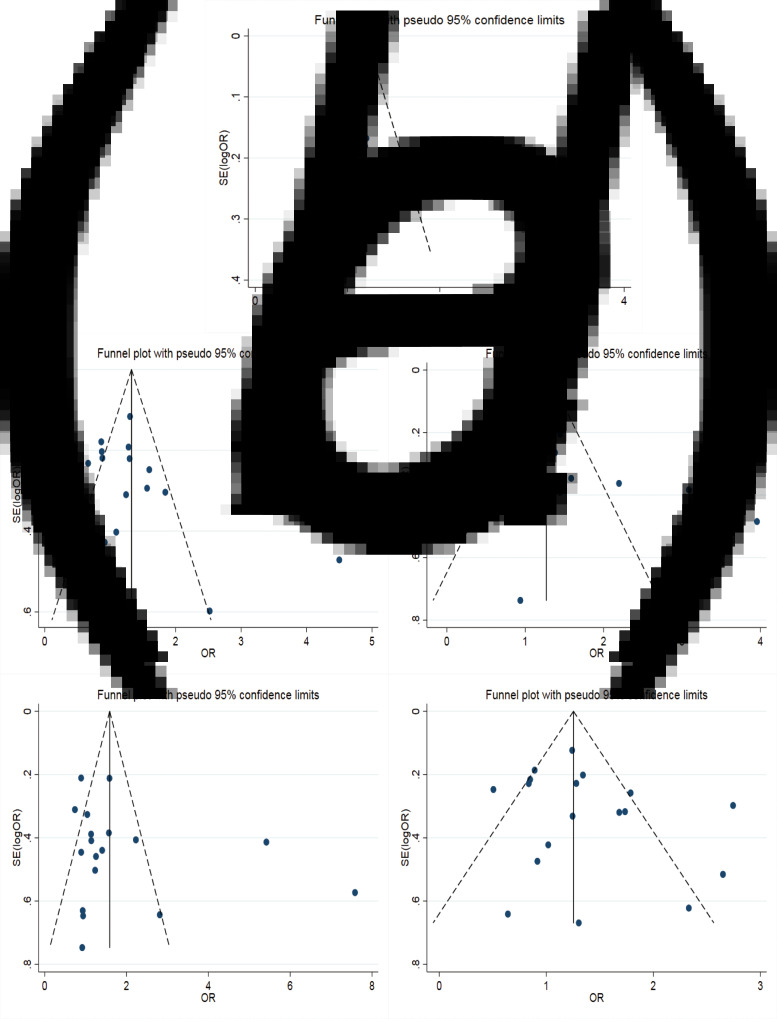
Funnel plot of IL-10 (rs1800871) polymorphism and susceptibility to HBV (a: allelic genetic model; b: dominant genetic model; c: recessive genetic model; d: homozygous genetic model; e: heterozygous genetic model).

The results of the meta-analysis of IL-10 rs1800871 polymorphism and the risk of HBV infection were found in Table 4 and Figure 3. Allele C compared with allele T, *I*^2^ = 60% (*P* < 0.05) indicates the existence of heterogeneity existed. The REM was used to calculate the pooled OR. The results showed that the pooled OR = 1.21 (95% CI 1.06–1.37, *P* < 0.01), the difference was statistically significant. The results of the subgroup analysis of the Asian population and the control group with a healthy population as controls were consistent with the overall analysis, the difference was statistically significant. Furthermore, heterogeneity decreased slightly. The forest plot was shown in Figure 3a. This suggests that the Allele C at IL-10 rs1800871 loci is associated with the risk of HBV infection. The funnel plot was basically symmetrical (Fig. 4a) and Egger's Test showed that *P* > 0.05 indicating no publication bias.

**Table 4.** Main results of meta-analysis of HBV susceptibility to IL10 rs1800871 polymorphisms

Genetic model	Subgroup	*n* of study	OR	95% CI	*p*	I2(%)	*P* for Heterogeneity	Model	*P* for Publication bias (Egger)
Allelic model (C *vs.* T)	Overall	18	1.21	1.06–1.37	0.004	60.0	0.001	REM	0.133
	Asian	14	1.16	1.01–1.32	0.031	56.0	0.006	REM	0.523
	Caucasian and African	4	1.52	0.99–2.32	0.055	71.9	0.014	REM	0.218
	Based on HC	15	1.16	1.02–1.31	0.021	52.7	0.009	REM	0.507
	Based on S	3	1.69	0.90–3.14	0.100	80.6	0.006	REM	0.390
Dominant model (CC + CT *vs.* TT)	Overall	18	1.28	1.04–1.56	0.017	60.8	0.000	REM	0.205
	Asian	14	1.22	0.99–1.49	0.056	61.3	0.001	REM	0.538
	Caucasian and African	4	1.80	0.81–3.97	0.147	59.7	0.059	REM	0.987
	Based on HC	15	1.20	0.99–1.47	0.063	58.8	0.002	REM	0.662
	Based on S	3	2.24	0.91–5.53	0.080	63.1	0.066	REM	0.733
Recessive model (CC *vs.* CT + TT)	Overall	18	1.20	1.06–1.37	0.005	33.6	0.082	FEM	0.163
	Asian	14	1.16	1.00–1.34	0.054	29.5	0.141	FEM	0.458
	Caucasian and African	4	1.38	1.05–1.80	0.020	49.3	0.116	FEM	0.185
	Based on HC	15	1.17	1.02–1.35	0.029	26.0	0.168	FEM	0.437
	Based on S	3	1.67	0.85–3.27	0.137	26.0	0.168	REM	0.389
Homozygous model (CC *vs.* TT)	Overall	18	1.40	1.07–1.83	0.013	50.6	0.007	REM	0.233
	Asian	14	1.30	1.00–1.69	0.054	43.7	0.041	REM	0.595
	Caucasian and African	4	2.13	0.82–5.49	0.119	67.1	0.028	REM	0.639
	Based on HC	15	1.27	1.07–1.52	0.011	40.0	0.055	FEM	0.671
	Based on S	3	2.75	0.85–8.88	0.090	73.1	0.024	REM	0.567
Heterozygous model (CT *vs.* TT)	Overall	18	1.21	0.99–1.47	0.062	54.9	0.003	REM	0.268
	Asian	14	1.18	0.96–1.46	0.117	60.5	0.002	REM	0.448
	Caucasian and African	4	1.39	0.83–2.32	0.208	29.6	0.235	FEM	0.800
	Based on HC	15	1.21	0.99–1.47	0.144	58.5	0.002	REM	0.613
	Based on S	3	1.63	0.92–2.88	0.093	18.8	0.292	REM	0.421

OR, odds ratio; CI, confidence interval; REM, random-effects model; FEM, fixed-effect model; HC, healthy control; S, self-limiting infection controls.

#### Dominant genetic model

Genotype CC + CT compared with genotype TT, *I*^2^ = 60.8% (*P* < 0.05), indicated that there was heterogeneity among studies. The REM was used to estimate the pooled OR. The results showed that the pooled OR = 1.28 (95% CI 1.04–1.56, *P* < 0.05), the difference was statistically significant. The subgroup analysis of the Asian population and the control group with healthy people as controls showed that the heterogeneity did not decrease significantly. Forest plot was shown in Figure 3b. The funnel plot was basically symmetrical (Fig. 4b) and Egger's Test showed that *P* > 0.05 showing no publication bias.

#### Recessive genetic model

Genotype CC compared with genotype CT + TT, *I*^2^ = 33.6% (*P* > 0.05), indicated that the heterogeneity among studies was low. The FEM was used to calculate the pooled OR. The results showed that the pooled OR = 1.20 (95% CI 1.06–1.37, *P* < 0.05) and the difference was statistically significant. The analysis of Asian population subgroup showed that the difference was not statistically significant and pooled OR = 1.16 (95% CI 1.00–1.34, *P* > 0.05), while the subgroup analysis of the controls based on healthy population showed that the difference was statistically significant, the pooled OR = 1.17 (95% CI 1.02–1.35, *P* < 0.05). Forest plot was found in Figure 3c. The funnel plot was basically symmetrical (Fig. 4c) and Egger's Test showed that *P* > 0.05 suggesting no publication bias.

#### Homozygous genetic model

Genotype CC compared with genotype TT, *I*^2^ = 50.6% (*P* < 0.05) indicated that the heterogeneity among the studies was statistically significant. The REM was used to calculate the pooled OR. The results showed that the pooled OR = 1.40 (95% CI 1.07–1.83, *P* < 0.05), the difference was statistically significant. The analysis of Asian population subgroup showed that the difference was not statistically significant and the pooled OR = 1.30 (95% CI 1.00–1.69, *P* > 0.05), while the subgroup analysis of the controls based on the source of the healthy population showed that difference was statistically significant and the pooled OR = 1.27 (95% CI 1.07–1.52, *P* < 0.05). The forest plot was shown in Figure 3d. The funnel plot was basically symmetrical (Fig. 4d) and Egger's Test showed that *P* > 0.05 indicating no publication bias.

#### Heterozygote model

Genotype CT compared with Genotype TT, *I*^2^ = 54.9% (*P* < 0.05) showed heterogeneity among studies. As a result, the REM was used to calculate the pooled OR. The results showed that the difference was not statistically significant, the pooled OR = 1.21 (95% CI 0.99–1.47, *P* > 0.05). The results of the subgroup analysis of the Asian population and the controls based on a healthy population were consistent with those of the overall analysis, which showed no statistical significance. Nevertheless, the heterogeneity did not decrease. The Forest plot was shown in Figure 3e. The funnel plot was basically symmetrical (Fig. 4e) and Egger's Test showed that *P* > 0.05 indicating no publication bias. This suggested that there was no correlation between the IL-10 rs1800871 heterozygote gene model and the risk of HBV infection.

### Sensitivity analysis

**Fig. 5.**
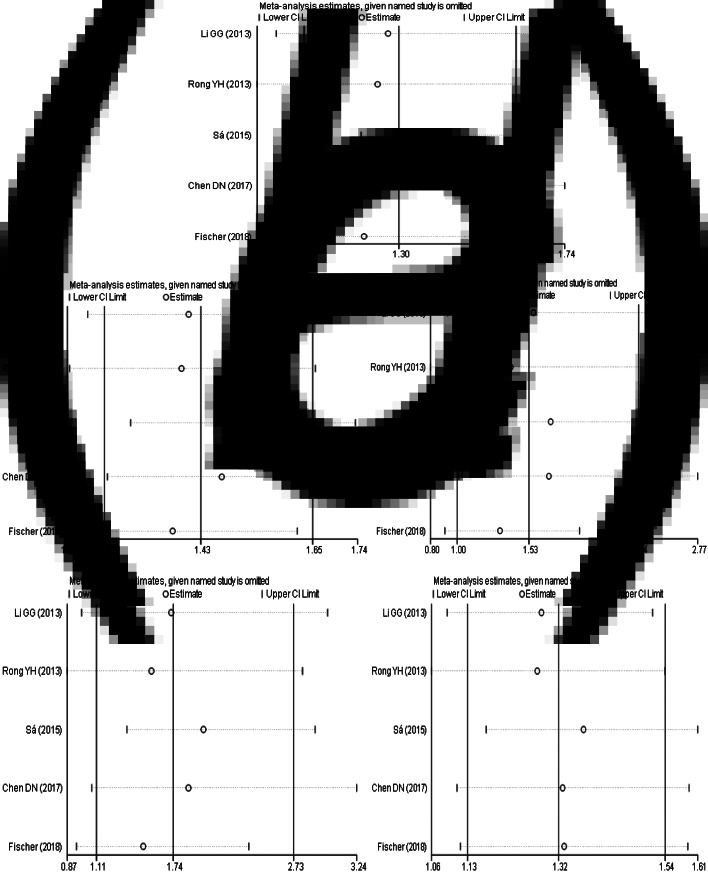
Sensitivity analysis of TLR3 (rs3775291) polymorphism and susceptibility to HBV (a: allelic genetic model; b: dominant genetic model; c: recessive genetic model; d: homozygous genetic model; e: heterozygous genetic model).

The results of sensitivity analysis (Fig. 5a–e) between TLR3 rs3775291 polymorphism and the risk of HBV infection showed that there was no statistically significant change in the findings of the dominant genetic model and heterozygous genetic model after removing any study, indicating that the conclusions of the two models were relatively robust. Allele model, recessive gene model and homozygous gene model, after removing some studies, the conclusions were different from the original, indicating that the findings of these three gene models should be cautious.

**Fig. 6.**
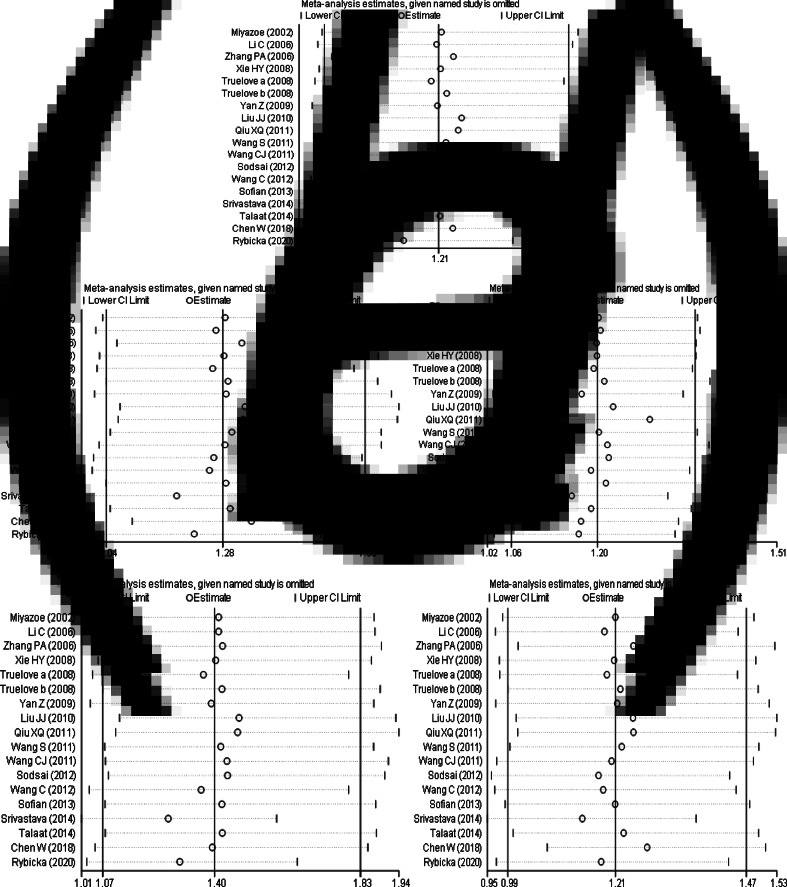
Sensitivity analysis of IL-10 (rs1800871) polymorphism and susceptibility to HBV (a: allelic genetic model; b: dominant genetic model; c: recessive genetic model; d: homozygous genetic model; e: heterozygous genetic model).

The results of sensitivity analysis (Fig. 6a–e) between IL-10 rs1800871 polymorphism and the risk of HBV infection showed that in the dominant genetic model (Fig. 6b), the difference was not statistically significant after the study of Srivastava *et al*. [17] was excluded. However, there was no statistically significant change in allele model (Fig. 6a), recessive model (Fig. 6c), homozygous model (Fig. 6d) and heterozygous model (Fig. 6e) after removing any study. Generally speaking, the conclusion was relatively robust.

## Discussion

The main purpose of this meta-analysis is to explore the correlation between TLR3 rs3775291 and IL-10 rs1800871 single nucleotide polymorphisms and the risk of HBV infection. Our meta-analysis showed that TLR3 rs3775291 and IL-10 rs1800871 gene polymorphisms were firmly related to the risk of HBV infection. However, the pathogenic mechanism of TLR3 and IL-10, affecting the risk of HBV infection, remains to be further studied. Zhang *et al*. [38] reported that TLR3 activation in hepatic stellate cell lines can inhibit HBV replication in HepG2 cells. TLR3 ligands could activate hepatocytes and immune cells, and antiviral cytokines can be produced by stimulated hepatocytes and peripheral blood monocytes [39]. TLR3 also plays an important role in HBV-related hepatocellular carcinoma [40]. The expression of TLR3 is related to apoptosis, proliferation and angiogenesis of hepatocellular carcinoma, which could predict the prognosis [7]. T cell activation stimulated by TLR3 can be inhibited by HBsAg, while HBsAg can be reversed by anti-IL-10 antibody [41]. This may be part of the reason why HBV evades innate and adaptive immune responses to maintain persistent infection. In chronic HBV infection, regulatory B cells that produce IL-10 inhibit effector T cells and enhance regulatory T cells [42]. Xu *et al*. [43] reported that IL-10 derived from Kupffer cells plays a key role in maintaining persistent humoral immune tolerance to the hepatitis B virus in mice. In patients with acute and chronic hepatitis B, the expression of IL-10 also affects their prognosis [44].

A meta-analysis of TLR3 rs3775291 and HBV infection risk showed that pooled OR was statistically significant in the allele model, dominant gene model, homozygous gene model and heterozygous gene model. In the recessive gene model, the pooled OR was not statistically significant. Due to the limited literature, only five articles were included, so the funnel plot was not used to test the publication bias. However, the results of Egger's Test showed that the *P-*value of each genetic model was higher than 0.05, indicating that the publication bias was well controlled. From the results of sensitivity analysis, the dominant gene model and heterozygous gene model had good robustness, while other genetic models had some instability. Therefore, we could believe a correlation between TLR3 rs3775291 single nucleotide variation and the risk of HBV infection. This study was the first systematic analysis of the relationship between TLR3 rs3775291 loci polymorphism and the risk of HBV infection. This could be said to be an important discovery and of great significance.

Meta-analysis of IL-10 rs1800871 and HBV infection risk showed that the pooled OR was statistically significant in allele genetic model, dominant genetic model, homozygous genetic model and recessive genetic model. In the heterozygous genetic model, the pooled OR was not statistically significant. Except for the small heterogeneity of the recessive gene genetic model, the heterogeneity of other genetic models is considerable, so we analysed the subgroup of Asian population and the subgroup of healthy population separately, but the heterogeneity did not decrease significantly. The funnel plots of all genetic models were basically symmetrical and Egger's Test also showed that their *P*-values were all greater than 0.05, indicating no obvious publication bias. Sensitivity analysis showed that the dominant gene genetic model and heterozygote model had good robustness, while other gene genetic models were unstable. The meta-analysis of Ren *et al*. [45] showed that allele C at the IL-10 rs1800871 loci and genotype CC + CT increased the risk of HBV infection in the Asian population with healthy controls. In contrast, genotype CC was not associated with the risk of HBV infection. The conclusions of the allele model and recessive gene model were consistent with our research. However, our results showed that the CC + CT genotype was not associated with the risk of HBV infection in the Asian population. This might be related to the fact that we had included more research.

Inextricably, this study also had some limitations. First of all, the number of studies included was limited, especially on the risk of TLR3 rs3775291 and HBV infection, only five articles included in this meta-analysis. The limited sample size might have a particular bias on the inference of the conclusion. Secondly, the Ethnicity included in the study had some limitations. In the study of the risk of IL-10 rs1800871 and HBV infection, most of the subjects were Asian, while those of other races were relatively fewer. Thirdly, the results of sensitivity analysis showed that there was a certain degree of instability between the risk of HBV infection and some genetic models of TLR3 rs3775291 and IL-10 rs1800871 loci. After some studies were excluded, the conclusion changed statistically. Finally, due to the limitations of the studies included, this meta-analysis did not make a further analysis of the interaction between gene and gene, gene and the environment.

In conclusion, there was a correlation between single nucleotide polymorphism at TLR3 rs3775291 and IL-10 rs1800871 loci and the risk of HBV. Allele C and genotype CC at IL10 rs1800871 loci and allele T and genotype TT at TLR3 rs3775291 loci may increase the risk of HBV infection. However, considering the limitations of this study, such as the small sample size of TLR3 rs3775291 loci and HBV infection, the robustness of IL-10 rs1800871 conclusions and the interaction between genes and genes, etc., it is necessary to continue to study the relationship between the above susceptible gene polymorphisms and susceptibility to HBV infection.

## Availability of data and material

The datasets used and/or analysed during the current study are available from the corresponding author on reasonable request.
